# Impact of promoting blood donation in general practice: Prospective study among blood donors in France

**DOI:** 10.3389/fpubh.2022.1080096

**Published:** 2022-12-06

**Authors:** Ophélie Renaux, Leila Bouazzi, Antoine Sanchez, Judith Hottois, Marie-Charlotte Martin, Jan Chrusciel, Stéphane Sanchez

**Affiliations:** ^1^French Blood Transfusion Service, Troyes, France; ^2^University Committee of Resources for Research in Health (CURRS), University of Reims Champagne-Ardenne, Reims, France; ^3^Private Hospital Burgundy Dijon, Dijon, France; ^4^General Practice Department, University of Reims Champagne-Ardenne, Reims, France; ^5^Pôle Territorial Santé Publique et Performance des Hôpitaux Champagne Sud, Centre Hospitalier de Troyes, Troyes, France

**Keywords:** blood donation, general practice, health promotion, waiting room, blood donor

## Abstract

**Introduction:**

Waiting rooms in general practitioners' (GP) surgeries are a potentially useful site for spreading educational messages about health behaviors. We aimed to evaluate the impact of posters displayed in GPs' waiting rooms on the number of donors attending the blood donation drives in the Aube Department of France. The secondary objective was to identify self-reported factors that incited people to give blood among donors who did and donors who did not see the posters.

**Methods:**

Observational, multicenter, prospective study, from 1 June to 31 December 2021. Six blood donation centers in the Aube Department were selected. All GPs located within a 15 km radius around each center were invited to participate by hanging posters advertising blood drives in their waiting rooms. The number of blood donations per hour was measured before and during the campaign. Factors prompting people to give blood were evaluated by questionnaires completed by persons attending the blood drives.

**Results:**

33 GPs participated. The number of donations per hour was lower in the year in which the posters were displayed (2021) compared to the previous year (12 vs. 15). A total of 1,469 questionnaires were completed by blood donors: 729 reported having seen the posters, and 740 reported not having seen the posters. Those who claimed to have seen the posters were more likely than those who claimed not to have seen the posters to respond that in parallel, they had been prompted to give blood via online publicity (7.5 vs. 3.9%, adjusted Odds ratio [aOR] 1.75, 95% confidence interval [CI] 1.12–2.82, *p* = 0.02). They also more often reported that they had been prompted to donate by television advertisements (8.0 vs. 4.2%, aOR 1.74, 95%CI 1.10–2.76, *p* = 0.02). Overall, 68% of all respondents indicated that posters in the GP's waiting room would incite them to give blood more often.

**Conclusion:**

The number of blood donations per hour was lower during the year in which posters were displayed. Questionnaire data from donors suggests that promoting blood donation *via* posters in GPs' waiting rooms could have a positive effect: 68% of donors claimed that posters would incite them to give blood.

## Introduction

France has a consistently high level of demand for blood, and demand rose steadily from 2003 to 2012 (1) due to medical progress combined with the increasing life expectancy of the French population. Demand for blood has been stable for the last 10 years (2). The French Blood Transfusion Service (BTS) under the supervision of the national Ministry of Health has been the sole organizer of blood drives and blood transfusion services in France since 1 January 2004. Its primary role is to ensure that France achieves self-sufficiency in the supply of blood products. In France, ten thousand blood donations per day are required to achieve self-sufficiency. To meet these needs, the BTS is constantly searching for new donors. In 2020, the BTS recorded around 1.5 million blood donors, of whom around 250,000 were new (first-time) donors (16%), for a total of 2.8 million blood donations.

A shortage of available blood may sometimes occur due to a decrease in donors or an increase in deferrals (3). Such shortages may also happen in the context of a public health crisis, like the COVID-19 pandemic (4, 5). Therefore, it is of vital importance to have adequate recruiting procedures for donors with various blood types and backgrounds (6, 7). This is especially true for countries in which donors do not receive any payment (such as France).

The factors that influence the willingness to give blood have been explored in several studies (8–16). The main reported barriers to blood donation were lack of time, fear of contracting disease, the idea that one may be unfit or ineligible to give blood, a lack of information, and a fear of needles. In parallel, the main motivators were reported to be altruism, proximity of the home or workplace to a blood donation center, incitation by a loved-one, and the possibility of having a health check-up at the same time. Indeed, in France, blood donors undergo systematic screening for certain infectious diseases (e.g., HIV, hepatitis), and this can be a motivating factor for some people.

General practitioners (GPs) play a key role in advising patients, and as such, contribute to public health efforts (17). Visits to a GP could be a good opportunity to raise awareness about blood donation (18).

The waiting room of a GP's practice is an ideal location to deliver information about health-promoting behaviors. The waiting room space needs to be appropriately organized (19, 20) using adequate tools to maximize the impact of the health messages delivered. In the literature, up to 80% of persons surveyed declared that they read the posters hanging in the GP's waiting room (21, 22), while 53% of respondents in one study said they would be interested in receiving further information. Posters can also provide an opening for discussion on subjects that the patient might not otherwise have addressed. Finally, in a context where consultations are often too short to address all problems, the poster does not infringe on the GP's time. We therefore hypothesized that posters in GPs' waiting rooms would be effective in increasing the number of donors at blood drives. The aim of this study was to evaluate the efficacy of posters promoting blood donation on the number of people attending blood drives in nearby localities. Our secondary objective was to assess the reasons prompting blood donation among those who reported having seen the posters in the GP's waiting room, as compared to other donors.

## Materials and methods

### Study design and population

We performed a multicenter, observational, prospective, before-and-after study from 1 June 2021 to 31 December 2021. We used data on blood drive attendance from the BTS at blood drives held from 1 January 2017 to 31 December 2021. We selected six blood donation centers in the Department of the Aube, in Eastern France, all with an expected attendance of more than 50 donors at each drive, and with blood drives organized at least every 2 months.

### Primary and secondary objectives

The primary objective was to compare the number of donors per hour presenting at the blood drives between the year of the poster promotion campaign and previous years. The secondary objectives were: to evaluate the number of donors having seen the advertising posters in their GP's waiting room, to study the characteristics of these donors (including reasons for donating blood), and to ascertain how they differed from donors not having seen the posters.

### Recruitment

Six locations were selected at random among all the blood drives organized in the Aube Department on a monthly or bimonthly basis, namely: Arcis-sur-Aube, Bar-sur-Seine, Brienne-la-Vieille, Nogent-sur-Seine, Romilly-sur-Seine, Aix-en-Othe/Fontvannes. We chose only blood drives in localities outside the city of Troyes (capital city of the Department), since we hypothesized that GPs practicing in rural and semi-rural areas would have more developed roles as family doctors. We invited all GPs practicing within a radius of 15 kilometers around the locality of the selected blood drives to participate in the study.

### Promotion of blood donation by participating GPs

Posters advertising the blood drives were displayed in the waiting rooms of participating GPs' surgeries. Participating GPs received two different types of poster both delivered by the lead researcher in May 2021.

The first type of poster (see Supplementary Figure 1) was in A3 format and aimed to promote blood donation among community dwellers. The poster was developed by the communication department of the Greater Eastern France division of the BTS. GPs were asked to display it in the waiting room of their practice for the entire duration of the study (7 months in total).

The second type of poster (see the example in Supplementary Figure 2), was in A4 format, and gave the date, time and location of the next upcoming blood drive in the nearest blood donation center (one of the 6 selected participating centers). These posters were sent to the GPs 3 weeks to 1 month prior to the blood drive.

### Questionnaire for blood donors

A questionnaire comprising seven questions (Appendix 1, Supplementary material) was distributed to all donors attending the blood drives. The questionnaire aimed to assess whether the donors had seen the advertising posters in the GP's waiting room. It also sought to identify the factors prompting people to give blood on that particular day.

### BTS blood donation drive attendance data

We investigated variations in attendance at the blood drives during the study period compared to previous years using data from the BTS. We extracted the number of donors attending each blood drive, as well as their sex, age, postal code of residence, and number of donations.

### Statistical analysis

Continuous variables are expressed as mean ± standard deviation for normally distributed variables, or as median and range for other quantitative variables. Categorical variables are expressed as number and percentage.

We recorded the total number of donors from 2015 to 2021, as well as the product of the number of hours the drive was open by the number of drives per site. To calculate the number of donors per hour for each site, the total number of donors per site was then divided by the number of hours the blood drive was open. The same calculation was performed for all the data on the questionnaire.

It should be noted that for the mobile units collecting blood at selected sites outside of major towns, there are usually two staff members welcoming blood donors and recording their medical information at arrival, whereas at the permanent blood transfusion center in the city of Troyes, there is only one person at the welcome desk. Therefore, the number of donors per hour was numerically lower in Troyes compared to other locations.

Continuous variables were compared using Student's *t* or Mann-Whitney *U* tests, as appropriate according to their distribution. Categorical variables were compared using the Chi-square test or Fisher's exact test, as appropriate. The mean number of donors per year was compared over the 5 years of BTS data using ANOVA.

All statistical analyses were performed using SAS version 9.4 (SAS Institute Inc., Cary, NC). A *p* < 0.05 was considered statistically significant.

## Results

**Figure 1:**
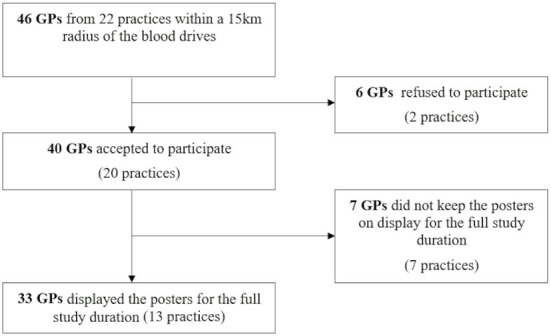
Flowchart of the selection of participating general practitioners.

A total of 33 GPs from 20 practices participated in the study (Figure 1). There were 30 blood drives held during the study period; the details are given in Table 1.

**Table 1:** Summary of the number of blood drives at each of the 6 selected sites during the study period.

**Location**	**Number of blood drives**
Arcis-sur-Aube	4
Bar-sur-Seine	5
Brienne-la-vieille	4
Nogent-sur-Seine	7
Romilly-sur-Seine	6
Aix-en-Othe/Fontvannes	4
TOTAL	30

**Figure 2:**
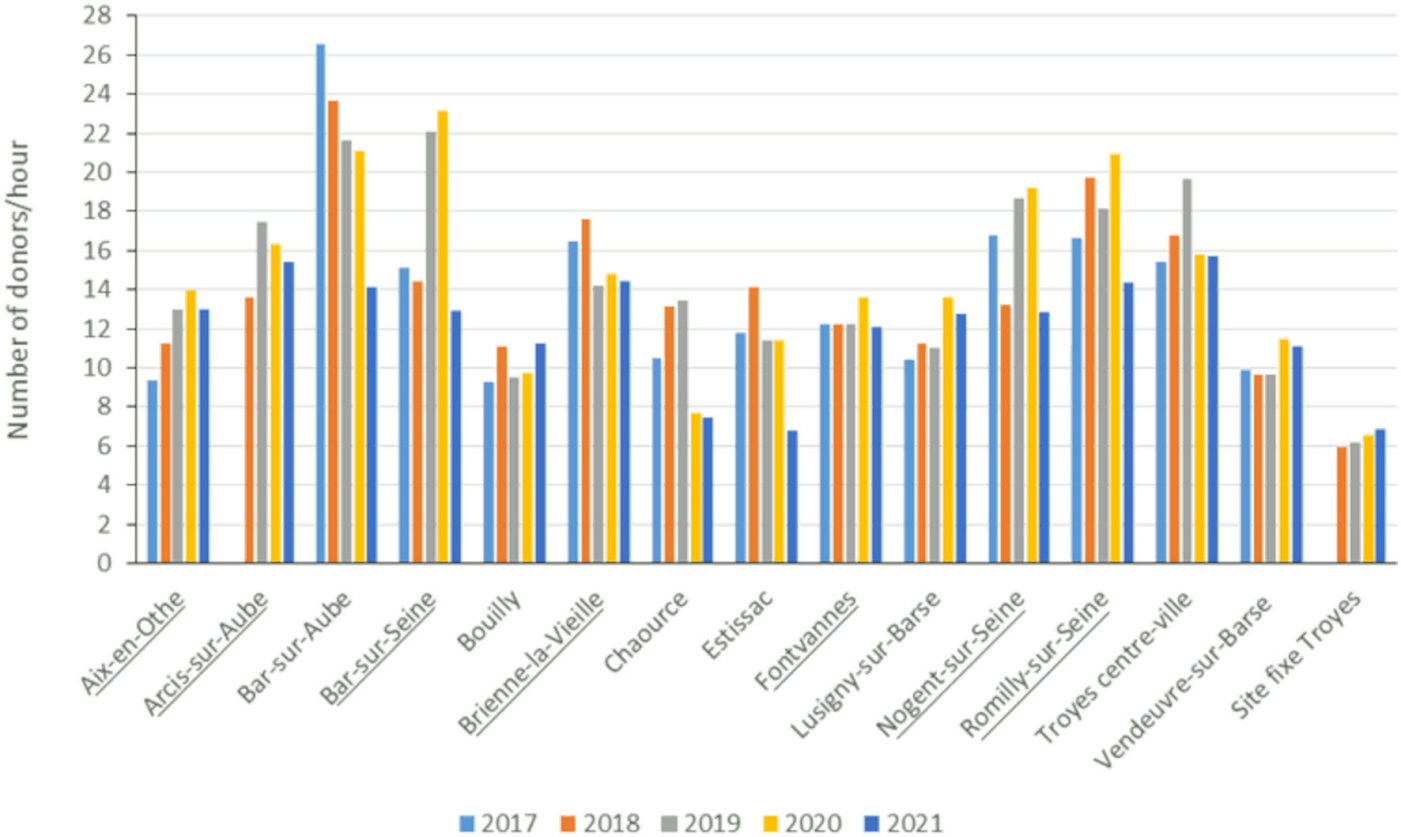
Trends in attendance at blood drives between 2017 and 2021, stratified by donation site. Data provided by the French Blood Transfusion Service (BTS). Underlined towns are the locations of the blood drives included in the study.

**Figure 3:**
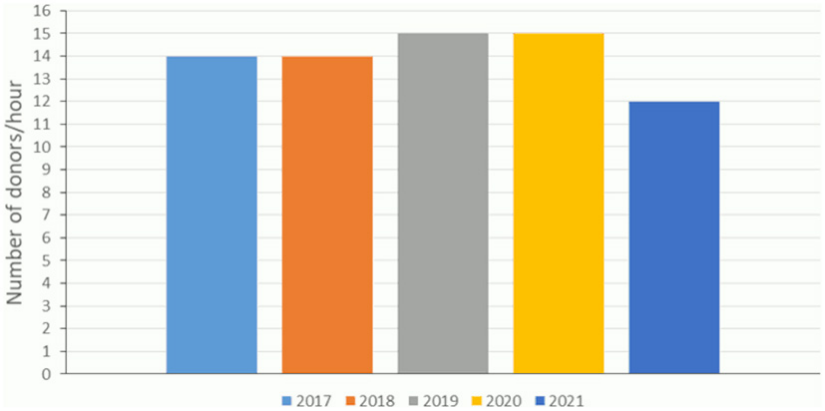
Trends in the average number of donors per hour in blood donation drives, from 2017 to 2021. Data provided by the French BTS.

Table 2 and Figure 2 show the attendance rates (donors per hour) at the different blood drive sites during the study. Considering all sites together, a small increase in the number of donors per hour was observed in 2019 and 2020 (from 14 to 15 donors/hour), then a decline in 2021 (12 donors/hour) (Figure 3).

**Table 2:** Evolution of the number of donors per hour between 2017 and 2021.

	**2017**	**2018**	**2019**	**2020**	**2021**	***P*-value**
Aix-en-Othe	9	11	13	14	13	
Arcis-sur-Aube	NA	14	17	16	15	
Bar-sur-Aube	27	24	22	21	14	
Bar-sur-Seine	15	14	22	23	13	
Bouilly	9	11	10	10	11	
Brienne-la-Vieille	16	18	14	15	14	
Chaource	10	13	13	8	7	
Estissac	12	14	11	11	7	
Fontvannes	12	12	12	14	12	
Lusigny-sur-Barse	10	11	11	14	13	
Nogent-sur-Seine	17	13	19	19	13	
Romilly-sur-Seine	17	20	18	21	14	
Site fixe Troyes	NA	6	6	7	7	
Troyes center-ville	15	17	20	16	16	
Vendeuvre-sur-Barse	10	10	10	12	11	
TOTAL	14	14	15	15	12	0.38

NA, no avalaible data. Results are presented by hour (for example 10 person per hours).

**Figure 4:**
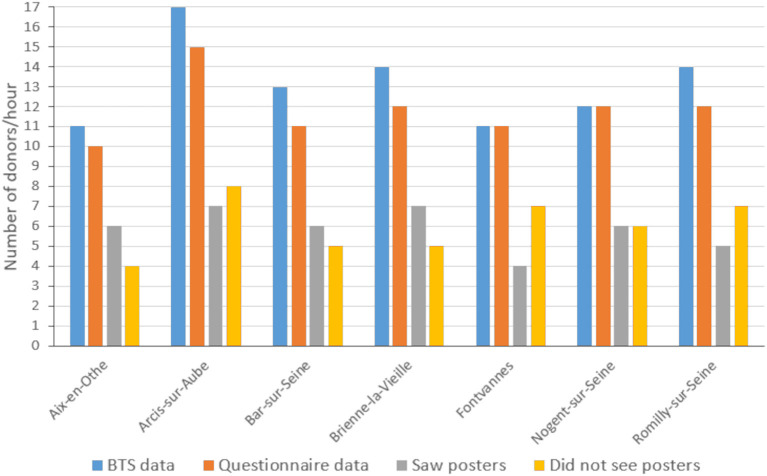
Total number of donors per hour attending the blood drives at each site during the study period, according to the BTS data (blue bars) and according to questionnaire data recorded on site by the study team (orange bars).

Overall, 1,469 blood donors completed the questionnaire during the blood drives held at the selected sites during the study period (88.8% of donors) (Table 3, Figure 4).

**Table 3:** Comparison of the number of donors per hour, and of those who had or had not seen the advertising posters in a GP's waiting room.

	EFS*	**Questionnaire**			
**Site of blood drive**	**Donors/hour**	**Donors/hour**	**Donors/hour who saw posters**	**Donors/hour who did not see posters**	***P*-value^a^**
Aix-en-Othe	11	10	6	4	0.79
Arcis-sur-Aube	16	15	7	8	
Bar-sur-Seine	12	11	6	5	
Brienne-la-Vieille	14	11	6	5	
Fontvannes	11	11	4	7	
Nogent-sur-Seine	12	11	6	5	
Romilly-sur-Seine	14	11	5	6	

^a^The p-value compares the results obtained among donors who saw the poster in the GP's waiting room and those who did not. EFS, French Blood Transfusion Service, GP, general practitioner.

Among these, 729 (49.6%) reported having seen an advertising poster in their GP's waiting room, while the remaining 740 donors (50.4%) reported not having seen the posters.

The characteristics of the two groups are presented in Table 4. There were no significant differences between those who claimed to have seen the posters and those who claimed not to have seen them, in terms of sex, age, and previous history of blood donation. The mean age was 45 years in both groups (*p* = 0.55). The percentage of women was 51.8% and 51.9% in the group that saw the posters and in the group that did not see the posters (*p* = 0.95). Among those who claimed to have seen the posters, 9.5% were first-time donors, compared to 7.1% in the group who had not seen the posters (*p* = 0.10). Among the 1,469 respondents, 518 were patients of GPs who were participating in the study. Of these 518 patients, 272 (52%) said they had seen the posters in their GP's waiting room.

**Table 4:** Univariate comparison of the characteristics of patients who saw the posters advertising the blood drive in a GP's waiting room, vs. those who did not.

	**Overall *N* = 1,469**	**Saw posters** ***N* = 729**	**Did not see posters *N* = 740**	***P*-value**
Age, years	45.3 ± 14.7	45.0 ± 15.1	45.5 ± 14.4	0.55
Sex				0.95
Female	761 (51.9)	375 (51.8)	386 (51.9)	
Male	706 (48.1)	349 (48.2)	357 (48.1)	
Not a first-time donor	1345 (91.7)	655 (90.5)	690 (92.9)	0.10
Reason for attending blood drive				
Accompanied/encouraged by a close friend or relative	132 (9.0)	72 (9.9)	60 (8.1)	0.21
Prompted by EFS television ads	89 (6.1)	58 (8.0)	3 (4.2)	0.002
Prompted by EFS advertisement on the internet	83 (5.7)	54 (7.5)	29 (3.9)	0.003
In response to an invitation sent by the EFS by text message	593 (40.4)	294 (40.7)	299 (40.2)	0.87
In response to an invitation sent by the EFS by e-mail	263 (17.9)	124 (17.1)	139 (18.7)	0.43
A friend or member of my family has a disease that requires blood transfusion	134 (9.1)	58 (8.0)	76 (10.3)	0.14
Opportunity: The blood drive is being held at a time when I'm free	709 (48.3)	354 (48.9)	355 (47.8)	0.67
Altruism: It's important to help people who are ill	1,097 (74.8)	549 (75.8)	548 (73.8)	0.36
I'm interested by the food provided after the blood donation	42 (2.9)	23 (3.2)	19 (2.6)	0.47
I'm interested by the blood test / health check-up provided	97 (6.6)	50 (6.9)	47 (6.3)	0.65
GP participating in the study	518 (35.3)	272 (37.6)	246 (33.1)	0.08
I believe that posters in the GP's waiting room would prompt me to give blood more often.	997 (68.0)	521 (72.1)	476 (64.1)	0.001

EFS, French Blood Transfusion Service, GP, general practitioner.

Overall, 987 participants (68%) said that educational posters in the GP's surgery promoting blood donation would prompt them to give blood more often; these participants were more frequently in the group that claimed having seen the posters (72.1%) than in the group that claimed not to have seen the posters (64.1%, *p* = 0.001).

By multivariable analysis (Table 5), blood donors who reported having seen the promotional posters in the GP's waiting room were significantly more likely to have been prompted, in parallel, to give blood by online publicity campaigns (OR 1.75, 95%CI 1.12–2.82, *p* = 0.02), or by television advertisements (OR 1.74, 95%CI 1.10–2.75, *p* = 0.02). The participation of the GP in the study was not significantly associated with increased odds of having seen the posters (OR 1.22, 95%CI 0.98–1.51, *p* = 0.08).

**Table 5:** Factors motivating participation in the blood drives.

	**Donors who saw the posters in a GP's waiting room *N* = 729**	**Donors who did not see the posters in a GP's waiting room *N* = 740**	**OR (95% CI)**	***P*-value**
Motivation for participation				
Saw a television advertisement	58 (8.0)	3 (4.2)	1.74 (1.10–2.76)	0.02
Saw an advertisement on internet	54 (7.5)	29 (3.9)	1.75 (1.12–2.82)	0.02
GP participating in study	272 (37.6)	246 (33.1)	1.22 (0.98–1.51)	0.08

GP, general practitioner; OR, odds ratio; CI, confidence interval.

The reasons motivating participants to give blood are detailed in Table 4. The most frequent motivating factors were altruism (*N* = 1,097; 74.8%), convenience/availability (the blood drive was on at a time when I was available) (*N* = 709; 48.3%), invitation received by text message (*N* = 593, 40.4%), invitation received by email (*N* = 263; 17.9%).

## Discussion

Contrary to what was expected, the attendance at blood drives did not increase in the year in which the posters were displayed by the GPs participating in the study. We observed an increase in attendance at blood drives in 2019 and 2020, compared to previous years, then a decline again in 2021. The surge in enthusiasm for blood donation corresponded to the timing of the SARS-CoV-2 pandemic. Therefore, the posters could have mitigated a decrease in blood donations caused by other factors, such as decreased interest in healthcare-related solidarity after the end of the first waves of the COVID-19 pandemic. In line with this hypothesis, the subjective responses of donors showed a positive appraisal of posters as a means of promoting blood donation: 68% said that the posters would encourage them to donate. Although posters were perceived to be useful, they were not sufficient to increase attendance at blood drives in the context of our study.

The self-reported factors motivating the participants in this study to donate blood were in line with previous reports in the literature. Altruism was the main motivator in our study and was cited by 74.8% of participants. Altruism is also the leading motivator in the literature, and was reported to motivate 50 to 90% of donors in various studies (10, 14, 23, 24). Being accompanied or encouraged by a close friend or family member was cited by 9% of our participants as their motivation. Higher rates have been reported elsewhere for this factor, sometimes exceeding one third of donors surveyed, mainly among first-time donors (10, 14, 24). Finally, as in other studies, we found that around 7% of participants were attracted by the opportunity of having a health check-up (14, 15). In France, for example, screening for infectious diseases is performed when people give blood (e.g., for HIV, Hepatitis B virus, Hepatitis C virus, and *plasmodium* species in case of recent travel to a country where malaria is endemic).

Studies have shown heterogeneous effects of delivering information in waiting rooms on the desired outcome. In one study (25), posters and pamphlets were declared to be the leading source of awareness about blood donation: 34% of students learned about blood donation from posters in an Indian study conducted with medical college students. Overall, the themes in posters promoting blood donation are surprisingly varied, although they often promote values of solidarity, feature the red color, and use international symbols (e.g., the red cross) (26). Television screens are increasingly popular, and studies have suggested that they have strong advertisement potential (27, 28). It has also been shown that in addition to the form of communication, the way the information is presented has a strong impact on the final quality of the message delivered (19, 20). Our results support the idea that messages delivered in waiting rooms can enhance awareness or knowledge albeit without systematically modifying behaviors (29).

Several messages were given precedence in the communication strategy adapted for the purposes of this study, namely the age group eligible to donate blood, and the main contraindications to blood donation, while also aiming to debunk the common myth that people who are taking treatment of any sort are ineligible for blood donation. In addition, the posters provided the practical information about when and where the next local blood drive would be held. It has also been shown that verbal incitation or digital reminders by text message or social networks could have a positive impact when the target population is well defined (30, 31).

In our study, 52.5% of donors whose GP was participating in the study reported that they had seen the posters promoting blood donation in their GP's waiting room. Our results differ in this respect from those in the literature (21), where 82% of patients reported having seen the posters. In the aforementioned study conducted in a group practice situated in Manchester, UK (21), those most likely to have seen the posters were aged over 50 years, and the probability of seeing the posters increased with the length of the wait. These discrepancies could be explained by the place where the questionnaire was administered, and the fact that some blood donors in good health may not have consulted their GP during the study period. There is also the question of the actual efficacy of the poster in making the patient decide to donate blood. The content of the poster could impact its efficacy; for example, messages emphasizing the negative consequences of the blood shortage for patients (loss-framed message) may have more impact in a crisis context (32). On the other hand, a message stating that donors can “save lives” does not always lead to more donations (33). The time frame expressed in the poster should match the time frame of the potential donor's intention (34). It has previously been reported that appropriate organization of the waiting room space is key in the delivery of health-promoting messages. In our study, there was no standardization regarding the placement of the posters in the waiting room, their position relative to other forms of information etc. Therefore, there may have been differences between participating GP surgeries in the practical implementation of the campaign. Finally, the manner of receiving and managing patients attending GP surgeries, and especially how to deal with waiting rooms and groups, has been profoundly affected by the SARS-CoV-2 pandemic. Many GPs' surgeries adapted hygiene measures such as asking patients to arrive at the exact time of their appointment, or to wait in their cars outside, with a view to limiting spread of the virus, but as a corollary, this also reduces the time during which people are exposed to health promoting messages.

Our study has some limitations. First, there may be potential for selection bias, as we did not record how many persons did not give blood. Second, the questionnaires were completed by the donors themselves, with the result that responses were undoubtedly subjective. Self-reporting is also prone to recall bias, whereby people may have forgotten that they saw the posters, or social desirability bias, whereby they give the answer that they think the researcher wants to hear. Third, we cannot exclude the presence of posters before the study period, which could have confounded the effect of the intervention. In the absence of any similar study in the literature, we were unable to calculate an appropriate sample size in advance. Future studies investigating trends in blood drive attendance and participation over more extended periods could provide more robust data about trends over time.

In conclusion, posters in the waiting rooms of GPs advertising upcoming blood drives do not appear to have a major effect on participation in blood drives, as an addition to the traditional media channels used for publicity by the French blood transfusion service (BTS). However, these posters could help to provide practical information to potential blood donors and likely reinforce the health behaviors already displayed by each individual. They do not seem to have a detrimental effect on attendance, and likely serve as reminders for people who already give blood regularly.

## Data availability statement

The original contributions presented in the study are included in the article and Supplementary material. Further inquiries can be directed to the corresponding author.

## Author contributions

OR, JC, and SS were involved in the conception and design of the study. SS, OR, M-CM, and JH were the coordinator of the study. OR were responsible for the data collection. OR and AS wrote the first draft. LB oversaw the analysis. OR, AS, JC, SS, and JH were involved in the interpretation of the study and critically reviewed the first draft. All authors approved the final version and accepted responsibility for the paper as published.

## Conflict of interest

The authors declare that the research was conducted in the absence of any commercial or financial relationships that could be construed as a potential conflict of interest.

## Publisher's note

All claims expressed in this article are solely those of the authors and do not necessarily represent those of their affiliated organizations, or those of the publisher, the editors and the reviewers. Any product that may be evaluated in this article, or claim that may be made by its manufacturer, is not guaranteed or endorsed by the publisher.
