# Supplementary material for: Impact of promoting blood donation in general practice: Prospective study among blood donors in France
Source: Front Public Health. 2022 Dec 6;10:1080096. doi: 10.3389/fpubh.2022.1080096 (PMC9763263; doi:10.3389/fpubh.2022.1080096)
Supplement: Supplementary file 1 [file Image_1.pdf]

Supplementary Material

Supplementary Figure 1. Blood Transfusion Service promotional poster displayed in General Practitioners' waiting rooms during the study [shown in French as in original version].

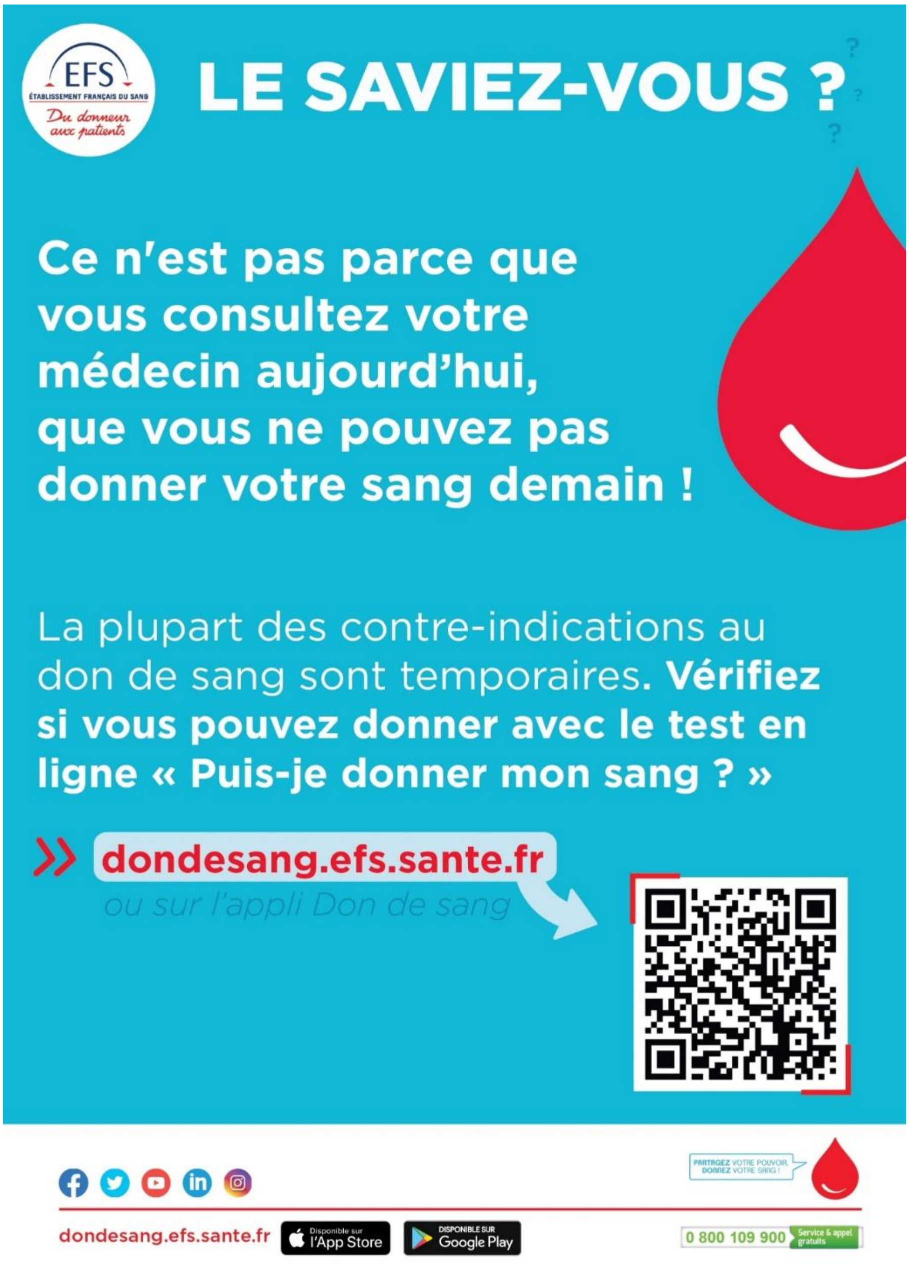

The main text of the poster can be translated as follows: " Did you know ? Just because you consulted your doctor today does not mean that you cannot donate blood tomorrow! Most contra-indications to blood donation are temporary. Check whether you can donate with the online test "Can I give blood ?""
