# Supplementary material for: Impact of promoting blood donation in general practice: Prospective study among blood donors in France
Source: Front Public Health. 2022 Dec 6;10:1080096. doi: 10.3389/fpubh.2022.1080096 (PMC9763263; doi:10.3389/fpubh.2022.1080096)

Supplementary Figure 2. Blood Transfusion Service memo poster with the dates of the next blood drive. This poster was displayed alongside the main promotional poster during the study [shown in French as in original version]

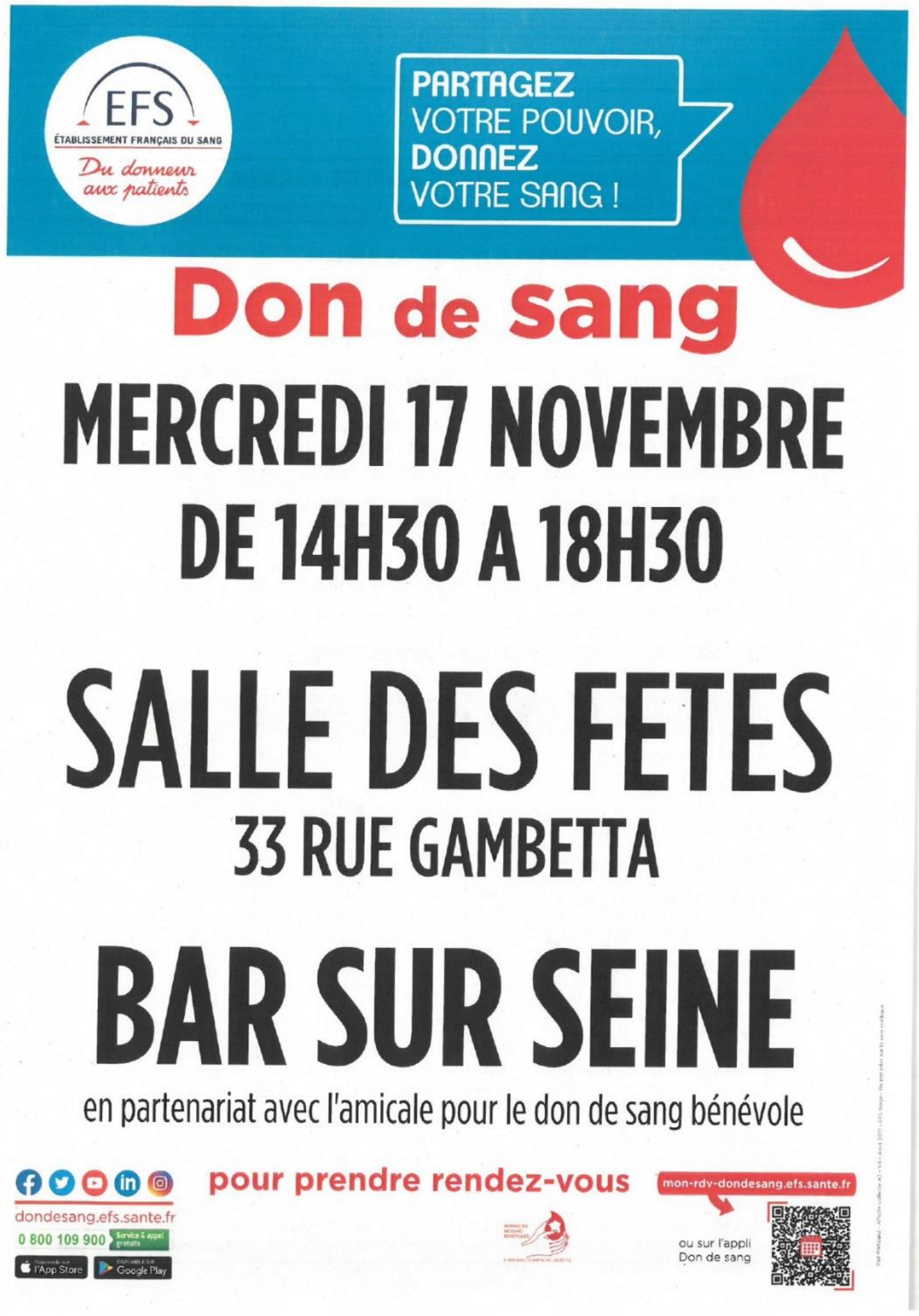

Supplement: Supplementary file 2 [file Image_2.pdf]
