# Supplementary material for: Impact of promoting blood donation in general practice: Prospective study among blood donors in France
Source: Front Public Health. 2022 Dec 6;10:1080096. doi: 10.3389/fpubh.2022.1080096 (PMC9763263; doi:10.3389/fpubh.2022.1080096)
Supplement: Supplementary file 3 [file Presentation_1.pdf]

Appendix 1. Questionnaire provided to blood donors during the study

A. English version, translated from French

Location: ____________________ Date: _____________________

Dear donor,

I am a ninth year general practice resident and I am conducting a study for my thesis, in partnership with the French Blood Transfusion service. I need your help to collect the highest possible number of responses to this questionnaire. This is an anonymous questionnaire that should only take you about two minutes. Through this questionnaire, I would like to ascertain what led you to give blood today.

This questionnaire is being distributed to all donors on arrival at the blood donation centre, for 6 months, from June 2021 to December 2021. Therefore, it is possible that donors may be asked to complete the questionnaire several times.

If you accept to respond to this survey, please be aware that your responses will be analyzed for the purposes of answering the question investigated in my thesis. Your answers are anonymous.

When you have finished completing the questionnaire (or if you do not wish to answer), please return it to the person at the welcome desk.

Thank you for your participation.

Ophélie RENAUX

Donor questionnaire (at collection site):

1. Are you: ☐ Male ☐ Female

2. What age are you? …………. years

3. Have you ever donated blood before? ☐ Yes ☐ No (first donation)

4. Who is your primary care doctor (GP):

Name: Dr …………………………….. Town: …………………………….

5. Have you ever seen a promotional poster regarding blood donation in your general practitioner's waiting room?

☐ Yes ☐ No

6. Do you think that seeing a promotional poster displayed in the waiting room of your general practitioner and a memo with the dates of blood collections scheduled near you would encourage you to donate blood more frequently ?

☐ Yes ☐ No

7. Why are you donating blood today?

You can tick one or more responses. If there are multiple responses please indicate their order of relevance by 1, 2, 3, …

☐ Incited / accompanied by a friend
☐ Incited by a televised advertisement campaign from the French Blood Transfusion Service
☐ Incited by an online advertisement campaign from the French Blood Transfusion Service

☐ Incited by an advertisement campaign from the French Blood Transfusion Service in the general practitioner's office
☐ Response to an invitation from the French Blood Transfusion Service by SMS text message
☐ Response to an invitation of the French Blood Transfusion Service by e-mail
☐ A member of your family / a friend has a disease for which transfusions are needed
☐ The blood drive was scheduled at a time when I was available
☐ It is important to help patients that are in need of a transfusion
☐ To have a free snack after the donation
☐ For the possibility of having blood tests done / health check-up

Ophélie RENAUX

B. Original version, in French

A :___________________________ Le : _________________

Chère donneuse, cher donneur,

Je suis étudiante en dernière année de médecine générale (interne en 9ème année d'étude) et je réalise un travail de thèse en relation avec l'Etablissement Français du Sang. J'ai besoin pour cela que vous acceptiez de m'aider afin de collecter un maximum de réponses à ce questionnaire. Il s'agit d'un questionnaire anonyme qui ne vous demandera que deux minutes. Je cherche, grâce à celui-ci, à connaitre vos motivations pour venir donner votre sang aujourd'hui.

Ce questionnaire est distribué à tous les donneurs à leur arrivée en collecte et ce pendant 6 mois, de juin à décembre 2021. Vous pouvez donc être amené à y répondre plusieurs fois.

Si vous acceptez de répondre à celui-ci, vous acceptez également que les réponses soient analysées afin de m'aider à répondre à ma question de thèse.

Une fois ce questionnaire rempli (ou si nous ne souhaitez pas y répondre), je vous prie de le retourner à l'agent d'accueil ou à la personne qui vous l'a remis.

Merci de votre participation. **Ophélie RENAUX**

Questionnaire donneur (en collecte) :

1. **Vous êtes :** ☐ Un homme ☐ Une femme

2. **Quel âge avez-vous ?** ………………… ans

3. **Avez-vous déjà donné votre sang ?** ☐ Oui ☐ Non (premier don de sang)

4. **Qui est votre médecin traitant ?** Nom : Dr ……………………………………… Ville : ………………………………………………

5. **Avez-vous déjà vu un affichage promotionnel pour le don du sang dans le cabinet de votre médecin généraliste ?**
   ☐ Oui ☐ Non

6. **Pensez-vous qu'un affichage promotionnel pour le don du sang ainsi qu'un rappel des dates de collectes près de chez vous dans la salle d'attente de votre médecin généraliste vous encouragerait à donner votre sang plus fréquemment ?**
   ☐ Oui ☐ Non

7. **Pour quelle raison donnez-vous votre sang aujourd'hui ?**

Possibilité de cocher une ou plusieurs réponses. Si plusieurs réponses merci d'indiquer l'ordre d'importance par 1,2,3, …

- ☐ Incité / accompagné par un ami
- ☐ Incité par une campagne publicitaire <u>télévisée</u> de l'Etablissement Français du sang
- ☐ Incité par une campagne publicitaire de l'Etablissement Français du sang sur <u>Internet</u>
- ☐ Incité par une campagne publicitaire de l'Etablissement Français du sang en <u>cabinet de médecine générale</u>
- ☐ En réponse à une invitation de l'Etablissement Français du sang par <u>SMS</u>
- ☐ En réponse à une invitation de l'Etablissement Français du sang par <u>e-mail</u>
- ☐ Un membre de votre famille / un ami à une maladie pour laquelle il bénéficie de transfusions sanguines
- ☐ La collecte de sang organisée près de chez vous correspond à un moment où vous avez le temps de donner
- ☐ Il est important d'aider les malades qui en ont besoin
- ☐ Accès à la collation après le don
- ☐ Possibilité de réaliser un bilan sanguin / bilan de santé

Ophélie RENAUX

<u>*Thèse de médecine générale :*</u> Impact de la promotion du don du sang en médecine générale – Etude prospective auprès des donneurs de sang de l'Aube.

A :___________________________ Le : _________________

Données personnelles

L'Université de Reims Champagne-Ardenne (Villa Douce -9, bd de la Paix -CS 60005 -51724 Reims Cedex), s'engage à ce que le traitement de données personnelles soit conforme au Règlement Général sur la Protection des Données (règlement UE 2016/679) et à la loi du 6 janvier 1978 modifiée (loi Informatique et Libertés).

Les données sont recueillies à des fins de recherche dans le cadre de la thèse « Promotion du don du sang en médecine générale : impact sur la fréquentation des collectes locales ».

La base légale du traitement de données est la mission d'intérêt public.

Les destinataires des données sont Ophélie RENAUX, interne réalisant sa thèse, Dr Judith HOTTOIS, en qualité de directrice de thèse et Dr Stéphane SANCHEZ, en qualité de responsable de médical de l'unité de recherche du centre hospitalier de Troyes.

Ces données sont conservées pour une durée de 2ans, le temps de la réalisation de la thèse.
Vous disposez d'un droit d'accès, de rectification et d'effacement des données qui vous concernent ainsi que d'un droit d'opposition et d'un droit à la limitation du traitement de ces données.
La fourniture des données est facultative.

Pour toute question sur le traitement de vos données, vous pouvez contacter Ophélie RENAUX à l'adresse suivante : ophelie.renaux@etudiant.univ-reims.fr

Si vous estimez, après avoir contacté la Déléguée à la Protection des Données de l'URCA à l'adresse suivante dpo@univ-reims.fr, que vos droits Informatique et Libertés ne sont pas respectés, vous avez la faculté d'introduire une réclamation à la CNIL, en ligne ou par courrier postal.
